# Evolution of compound eye morphology underlies differences in vision between closely related *Drosophila* species

**DOI:** 10.1186/s12915-024-01864-7

**Published:** 2024-03-19

**Authors:** Alexandra D. Buffry, John P. Currea, Franziska A. Franke-Gerth, Ravindra Palavalli-Nettimi, Andrew J. Bodey, Christoph Rau, Nazanin Samadi, Stefan J. Gstöhl, Christian M. Schlepütz, Alistair P. McGregor, Lauren Sumner-Rooney, Jamie Theobald, Maike Kittelmann

**Affiliations:** 1 Department of Biological and Medical Sciences, Oxford Brookes University, Oxford, OX3 0BP UK; 2 Integrative Biology and Physiology, UCLA, Los Angeles, CA 90095 USA; 3 Molecular Evolution and Systematics of Animals, Institute of Biology, University of Leipzig, Talstrasse 33, 04103 Leipzig, Germany; 4 Institute of the Environment and Department of Biological Sciences, Florida International University, Miami, FL USA; 5 Diamond Light Source Ltd, Harwell Science and Innovation Campus, Didcot, UK; 6 Swiss Light Source, Paul Scherrer Institute, Forschungsstrasse 111, 5232 Villigen PSI, Switzerland; 7 Department of Biosciences, Durham University, South Road, Durham, DH1 3LE UK; 8 Museum Für Naturkunde, Leibniz Institute for Evolution and Biodiversity Research, Berlin, 10115 Germany

**Keywords:** *Drosophila*, Eye size, Evolution, Ommatidia, Vision, Acuity, Contrast, Field of view, Optic lobe

## Abstract

**Background:**

Insects have evolved complex visual systems and display an astonishing range of adaptations for diverse ecological niches. Species of *Drosophila melanogaster* subgroup exhibit extensive intra- and interspecific differences in compound eye size. These differences provide an excellent opportunity to better understand variation in insect eye structure and the impact on vision. Here we further explored the difference in eye size between *D. mauritiana* and its sibling species *D. simulans.*

**Results:**

We confirmed that *D. mauritiana* have rapidly evolved larger eyes as a result of more and wider ommatidia than *D. simulans* since they recently diverged approximately 240,000 years ago. The functional impact of eye size, and specifically ommatidia size, is often only estimated based on the rigid surface morphology of the compound eye. Therefore, we used 3D synchrotron radiation tomography to measure optical parameters in 3D, predict optical capacity, and compare the modelled vision to in vivo optomotor responses. Our optical models predicted higher contrast sensitivity for *D. mauritiana*, which we verified by presenting sinusoidal gratings to tethered flies in a flight arena. Similarly, we confirmed the higher spatial acuity predicted for *Drosophila simulans* with smaller ommatidia and found evidence for higher temporal resolution.

**Conclusions:**

Our study demonstrates that even subtle differences in ommatidia size between closely related *Drosophila* species can impact the vision of these insects. Therefore, further comparative studies of intra- and interspecific variation in eye morphology and the consequences for vision among other *Drosophila* species, other dipterans and other insects are needed to better understand compound eye structure–function and how the diversification of eye size, shape, and function has helped insects to adapt to the vast range of ecological niches.

**Supplementary Information:**

The online version contains supplementary material available at 10.1186/s12915-024-01864-7.

## Background

Insect compound eyes exhibit remarkable variation in size as a result of differences in the number and diameter of the individual subunits, known as ommatidia. For example, the silverfish *Tricholepidion gertschi* has ~ 40 ommatidia [1] whereas dragon- and damselflies (Odonata) sport up to 30,000 ommatidia [2]. Differences in eye size as well as the number, size, and angles between facets allow different visual behaviours, lifestyles, and adaptation to a large range of environments [3–13]. How these diverse eyes of insects have evolved and to what extent even small changes in the optics affect vision is still not well understood. Investigating and comparing natural variation in eye size and composition, and its impact on optical capacity within and between closely related species can provide valuable insight into the functional evolution of the insect eye. Generally, wider ommatidia can harvest more light, allowing greater sensitivity, while more ommatidia and narrower interommatidial angles (IOs) can provide greater acuity [4, 14, 15]. Ommatidia diameter and number therefore represent a trade-off that is optimised for the specific visual needs of each insect species, strain, sex, or morph.

Several studies have reported the extensive variation in ommatidia number, ommatidia diameter, and overall eye size within and between species of *Drosophila* [16–21]. There is evidence that some of this is the result of trade-off between eye size and antennal size and, by extension, the visual and olfactory systems, as well as overall head capsule size [19, 20, 22]. The genetic basis of these differences in eye size is complex but, in some cases, the underlying genes and developmental mechanisms have been characterised [17, 20, 23–26]. We previously found that one *D. mauritiana* strain has larger eyes than its sibling species *D. simulans* as a result of wider ommatidia, potentially caused by differential expression of the transcription factor Orthodenticle (Otd) during eye development [17, 21, 26]. The larger eyes of *D. mauritiana* with respect to *D. simulans* are also associated with reciprocal changes in the distance between the eyes (face width), but the antennae were not examined [17].

Optical parameters can also vary within eyes: killer flies, including *Coenosia attenuata*, have evolved specialised wide frontal ommatidia with small IOs for diurnal aerial hunting [8], and males of many families of dipterans have enlarged ommatidia in the dorso-frontal region of the eye that allows them to detect and pursue females in flight [27]. *D. mauritiana* and *D. simulans* also exhibit structural variation across the eye, with significantly wider anterior frontal ommatidia than central and posterior ommatidia in both males and females [26].

However, to fully understand the functional impact of eye size variation within and between *Drosophila* species, it is crucial to test predictions based on eye morphology in vivo. Here we further explored the variation in ommatidia number and diameter that contribute to eye size differences in males and females among different *D. mauritiana* and *D. simulans* strains. We used synchrotron radiation microtomography to obtain 3D information of optical parameters in focal strains of these two species and tested predicted differences in their vision via optomotor responses in a virtual reality flight simulator in vivo.

## Results

### Ommatidia number and size vary within and between closely related Drosophila species

**Fig. 1:**
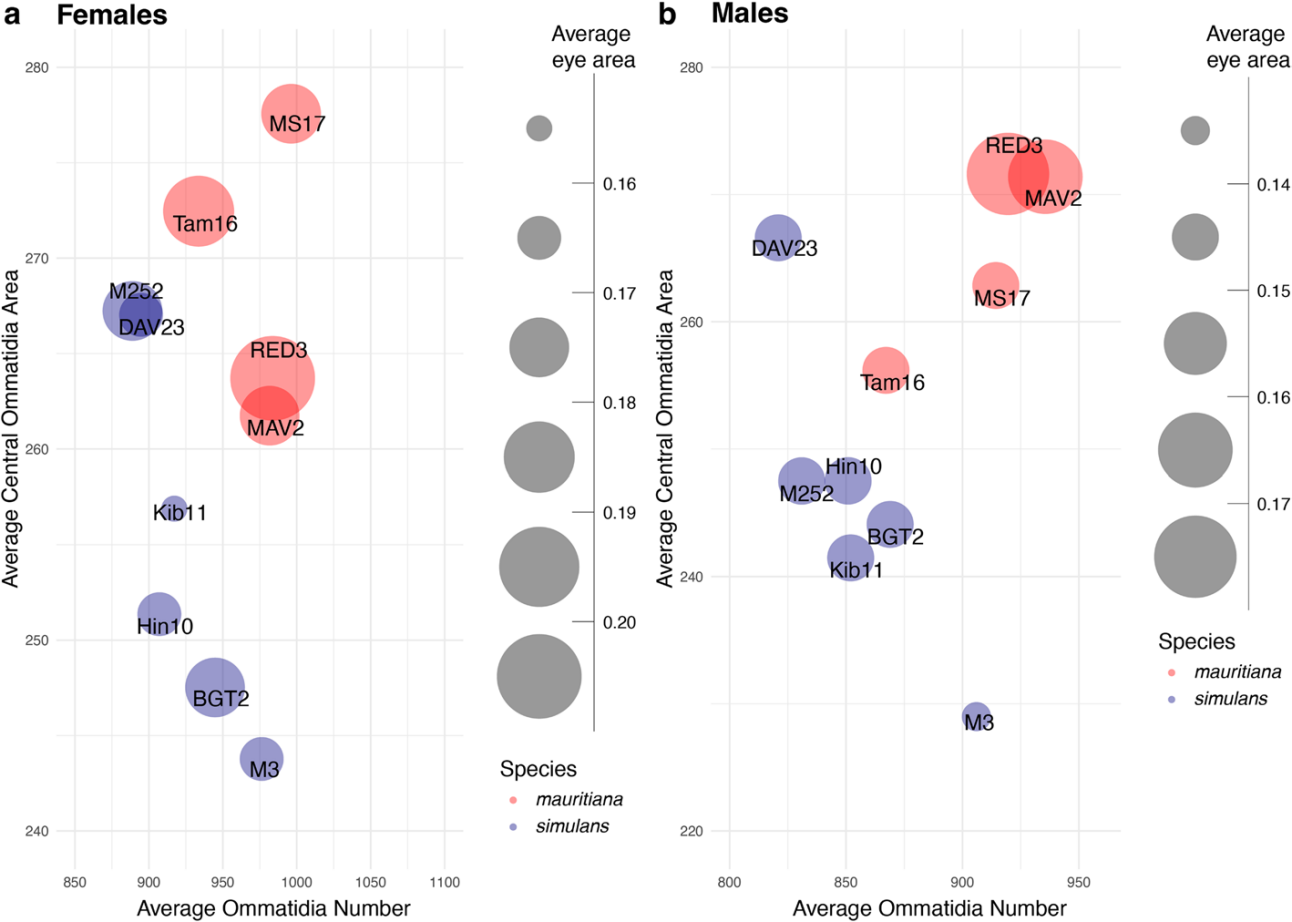
Variation in eye size, ommatidia number, and ommatidia size across closely related *D. mauritiana* and *D. simulans.* Average eye size (mm^2^, circle area) of *D. simulans* (blue) and *D. mauritiana* (red) strains (circle labels) plotted against total ommatidia number and ommatidia facet area (in μm.^2^) for females (**a**) and males (**b**). *D. mauritiana* generally have larger eyes with more and larger ommatidia compared to *D. simulans*. Eye size was measured from side view scanning electron micrographs of single eyes. *n* = 11 for MS17 females and *n* = 15 for males and females of all other strains. Measurements provided in Fig. 1 morphological measurements.xlsx on figshare [29]

**Fig. 2:**
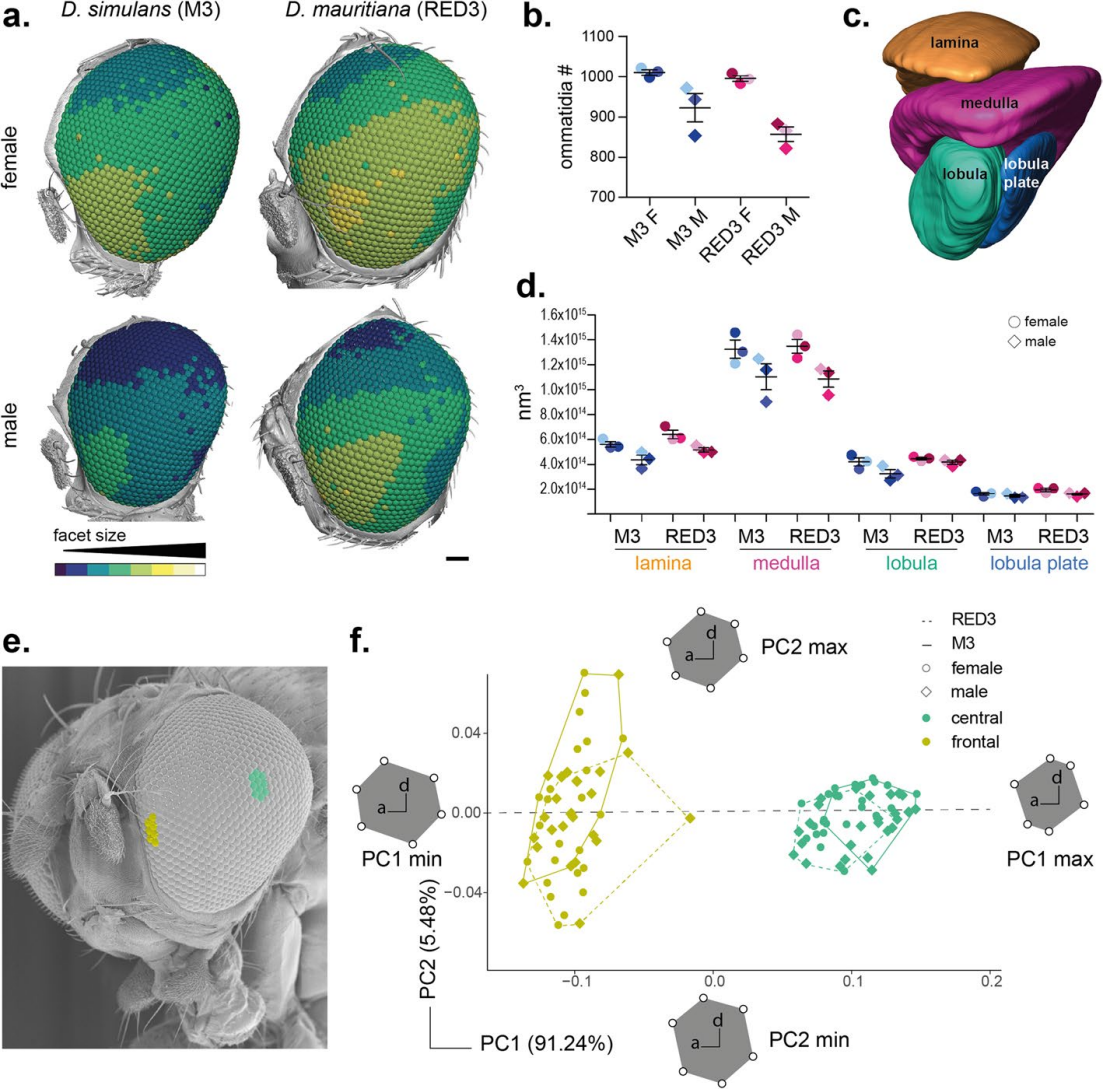
3D analysis of ommatidia size and shape in male and female *D. simulans* M3 and *D. mauritiana* RED3. **a** Synchrotron radiation microtomography analyses of males and females show a gradient from smaller to larger ommatidia from dorsal to anterior-ventral. *D. simulans* M3 females and especially males show an overall shift to smaller ommatidia compared to *D. mauritiana* RED3. **b** Analysis of ommatidia numbers show similar ommatidia numbers in females of both species and fewer ommatidia in males in line with overall smaller eye size. **c** 3D reconstruction of the optic lobe (lamina, medulla, lobula, and lobula plate) of a male *D. mauritiana* RED3. **d** Volume analysis of optic lobe neuropil sizes show a similar pattern to ommatidia number differences (**b**) between sexes and strains: males of *D. simulans* M3 *and D. mauritiana* RED3 have generally smaller neuropils, most evident in lamina and medulla (*n* (**a–d**) = 3). **e, f** Shape analysis of frontal (yellow) and central (green) ommatidia reveals separate clustering of frontal and central ommatidia for both species. *n* = 30. Data points are provided in Fig. 2 synchrotron analysis.xlsx and Fig. 2 morphometric analysis.xlsx on figshare [29]

The overall eye size of compound eyes is determined by ommatidia number and size (here reflected by facet area). While *D. mauritiana* generally have larger eyes than the closely related species *D. simulans* [16, 21, 28], it remains unclear if this difference is always caused by one or both parameters. We analysed total eye area, central facet area, and total ommatidia number from scanning electron microscopy images in multiple strains of both species (Additional File 1: Table S1) and found a negative correlation between central ommatidia facet size and number in *D. simulans* (females *R* =  − 0.38 *p* = 0.00021; males *R* =  − 0.3 *p* = 0.0036) suggesting a potential trade-off between these characteristics (Fig. 1, Additional File 2: Fig. S1). In contrast, *D. mauritiana* had generally wider and more numerous ommatidia and consequently overall larger eyes than *D. simulans* and the trade-off seen in *D. simulans* was absent in females. Interestingly, *D. mauritiana* males showed a positive correlation between ommatidia number and facet size (*R* = 0.34, *p* = 0.0087; Fig. 2, Additional File 2: Fig. S1).

To test whether larger eyes of *D. mauritiana* were an effect of overall larger body size, we also measured second-leg tibia length, which have been previously used as a proxy for body size [16, 30, 31] and the length of the L3 wing vein as an estimate of overall wing size [32]. The tibia of *D. mauritiana* were not generally larger than the tibia of *D. simulans* (Additional File 3: Fig. S2), suggesting the increase in eye size has evolved independently of body size [16, 17, 28]. Consistent with this, tibia size was only positively correlated with eye size in a subset of strains in both species (Additional File 4: Fig. S3). Interestingly, wing size is generally smaller in *D. mauritiana* strains, and we found strain-specific positive, negative, or no correlation with eye size (Additional File 5: Fig. S4).

While some *D. simulans* and *D. mauritiana* strains overlap in either ommatidia area or number, none of the strains overlapped in both parameters, leading to the clear separation of the species in eye composition (Fig. 1). Previously, a large-effect quantitative trait locus has been identified that explains about 30% of the eye size difference between *D. simulans* and *D. mauritiana* [17, 26] due to differences in ommatidia area. However, the functional consequences for vision in these flies remain unknown. To test this, we selected strains *D. simulans* M3 and *D. mauritiana* RED3 which have very similar ommatidia numbers but significantly different mean ommatidia (facet) areas (Fig. 1). We first performed detailed 2D and 3D morphological analysis of optical parameters of these focal strains to model their vision, and subsequently tested our predictions with behavioural experiments.

### Facet size and shape change in a dorsal to ventral-anterior gradient across species and sexes

*Drosophila* compound eyes are 3D structures that are roughly shaped like a hemisphere. To analyse optical parameters across the entire eye, we used synchrotron radiation microtomography to collect high-resolution 3D image data of entire eyes and associated brain structures in *D. simulans* M3 and *D. mauritiana* RED3 (Fig. 2). Automated segmentation and measurement of individual facets for three individuals of each species and sex revealed a size gradient from smaller dorsal-posterior to larger anterior-ventral facets in both focal strains and both sexes (Fig. 2a). Facet size was overall smaller in *D. simulans* M3 and the size difference between females and males was more pronounced in *D. simulans* M3 compared to *D. mauritiana* RED3 (Fig. 2a). Comparison of ommatidia number differences and neuropil volumes indicates similar patterns between sexes and strains. Lamina and medulla in males are generally smaller in line with lower ommatidia numbers (Fig. 2b–d).

Additionally, we used geometric morphometric analysis of facet shapes to compare central ommatidia to frontal ommatidia in both sexes of *D. simulans* M3 and *D. mauritiana* RED3 (Fig. 2e, f): the six corners of each facet were landmarked and analysed via principal component and hierarchical clustering analysis. We recovered three clusters, which can be interpreted as three distinct facet shapes. Clusters 1 and 2 contained only frontal lenses, and cluster 3 contained only central lenses indicating that the position of the facet within the eye influences lens shape. Frontal lenses (clusters 1 + 2) were defined by longer dorsal and ventral edges (PC1 = 87.3% variation) than central lenses (cluster 3). Within the frontal lenses, PC2 (4.5% variation) and PC9 (< 1% variation) separated clusters 1 and 2, with cluster 1 being slightly elongated along the antero-posterior axis. There were no differences in sex (chi-square = 0.07, df = 2, *p* = 0.967) or strain (chi-square = 2.74, df = 2, *p* = 0.254) between clusters, implying that these factors do not influence facet shape.

### D. mauritiana RED3 has greater optical sensitivity than D. simulans M3, especially in the frontal and ventral visual field

**Fig. 3:**
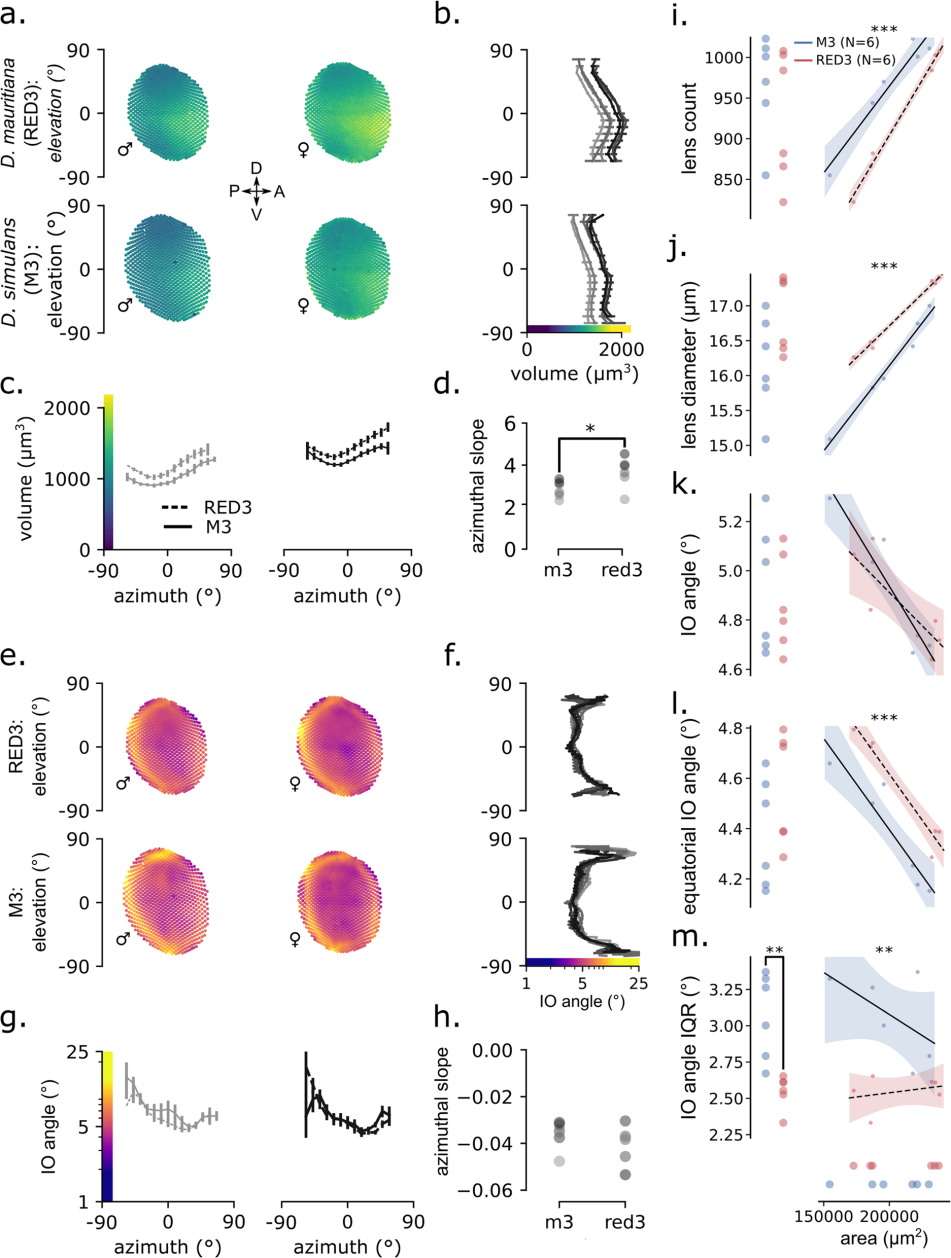
3D analysis of optic parameters in *D. simulans* and *D. mauritiana* eyes. **a–d** Ommatidial lens volumes from 3 males and 3 females from each of the two species. Eye maps show the smallest and largest eye of each of the two species, which also happen to be one male and female of each. Each dot of the scatter plot represents the location of an individual ommatidium in polar coordinates coloured by its 3D volume according to the colour scale indicated in the *x*- and *y*-axes of **b** and **c**. Line colours in **b** and **c** and dot colours in **d** indicate the fly’s rank in order of eye size per species, such that the darkest one is the largest eye of that species. The volume data is divided into 20 evenly spaced bins of elevation (**b**) and azimuth (**c**) with error bars indicating 3 times the standard error of the mean. **d** Ordinary least squares were used to regress lens volume on azimuthal position to estimate and compare the azimuthal slope of lens volume between the two species. The resulting slope coefficients from those models are plotted. **e–h** IO angle from three males and females from each of the two species, plotted as in **a–d** except for the elevation plot in **f**. The IO angle value used for each lens represents the average IO angle between that lens and all immediate neighbours. **f** The same IO angle data from **e** but sampling ommatidia from a narrow vertical band between 0 ± 15° azimuth. Note that this is different from **b**, **c**, and **g** because plotting the binned averages obfuscates the horizontal band of high acuity along the equator, likely due to the large range of IO angles along azimuth. **h** Ordinary least squares was used to regress IO angle on azimuthal position as in **d.** All the eyes demonstrated negative azimuthal slopes, with no significant difference between species. **i–m** Scatterplots of total lens count (**i**), mean lens diameter (**j**), median IO angle (**k**), median equatorial IO angle (**l**), and IO angle interquartile range (**m**) plotted along the *y*-axes and their allometric relationship to the surface area of their eye along the *x*-axis. Lines in the 2D scatter plots represent the predicted mean and the bands represent the 95% CI of the mean based on ordinary least squares regression of each *y* variable on surface area. Note that simple group differences based on ANOVA are indicated in the left margins and group differences after accounting for surface area using linear regression are indicated at the top of each scatterplot with the following key: * = *p* < .05, ** = *p* < .01, and *** = *p* < .001. Data provided in Fig. 3_share.zip on figshare [29]

To compare the optical capacity of both fly strains and their variation across the visual field, we implemented the open-source Python-based automated pipeline ODA [33], which estimates the location and approximate orientation of each lens with high resolution across the eye. This generated eye maps of the volume (Additional File 6: Fig S5a), diameter (Additional File 6: Fig S5b), cross-sectional area (Additional File 6: Fig S5c), and length (Additional File 7: Fig S6a) of the corneal lenses of the ommatidia and the mean IO angle of each lens with its nearest neighbours (Additional File 7: Fig S6b). Three male and three female eyes were scanned from both *D. simulans* M3 and *D. mauritiana* RED3. The coordinates were rotated manually to align the eye equators horizontally, visible as a horizontal band of smaller ommatidia in Fig. 3a (and Additional File 7: Fig. S6a, b, and c). This area projects roughly onto the visual horizon during flight [34] and marks the region of the eye where rows of ommatidia initiated and grew during eye development, establishing a line of mirror symmetry about which rhabdomere arrangements flip vertically [35]. To compare the change in these parameters from the posterior to the anterior eye, we used ordinary least squares to fit an affine function of azimuth and compared the resulting slope parameters for each subject.

Both *D. simulans* M3 and *D. mauritiana* RED3 eyes have the largest lenses in the frontal visual field just below the eye equator (Fig. 3a). For both species, lens volume increases with elevation, peaks just below the eye equator, and then decreases steadily (Fig. 3b). In *D. mauritiana* RED3 this increase is greater, starting at similar volumes at the dorsal and ventral extremes but increasing to larger maxima near the equator than *D. simulans* M3. Lens volume for both species decreases along elevation until a minimum around − 45° and then increases, peaking at the anterior extreme (Fig. 3c). Moreover, in 5 of the 6 size-ordered pairs, *D. mauritiana* RED3 have significantly greater lens volumes than *D. simulans* M3 for every azimuthal bin (Additional File 6: Fig S5a). Lens volume for all eyes has a positive azimuthal slope, but the slope for *D. mauritiana* RED3 was significantly greater than *D. simulans* M3 (*t*(10) = 2.3, *d* = 1.5, *p* = 0.043). This is consistent with measurements of lens diameter (Additional File 6: Fig S5b), cross-sectional area (Additional File 6: Fig S5c), and length (Additional File 7: Fig S6a), except that the azimuthal slope only differed for lens diameter (*t*(10) = 2.3, *d* = 1.4, *p* = 0.047) but not for either cross-sectional area (*t*(10) = 1.4, *d* = 0.9, *p* = 0.18) or length (*t*(10) = 1.9, *d* = 1.2, *p* = 0.08). Overall, this means that *D. mauritiana* RED3 have larger, broader, longer, and wider-spread ommatidial lenses than *D. simulans* M3, which could improve sensitivity in general, and especially in the frontal visual field below the eye equator. This increase in ventral optical sensitivity is also greater in *D. mauritiana* RED3 than *D. simulans* M3 and is predicted to improve the detection of low-contrast objects below the visual horizon, such as rotting fruit or other oviposition sites.

### D. mauritiana RED3 and D. simulans M3 have higher spatial acuity along the eye equator

IO angles are largest at the posterior and peripheral extremes, reaching a minimum around 45° azimuth along the eye equator (Fig. 3e, f). For both species, IO angle stays relatively constant—remaining between 4° and 6° from about − 45° to 45° elevation—except for dramatic increases at the ventral and dorsal extremes and a region of smaller IO angles around the eye equator (Fig. 3f). *D. mauritiana* RED3 ranges less in IO angle than *D. simulans* M3 reaching smaller maxima in the top and bottom of the eye (≤ 15° versus ≤ 25°). For both species, IO angle decreases along azimuth from a maximum in the posterior extreme (≤ 15°) to a minimum around 45° azimuth (≥ 4°; Fig. 3g). We found no significant difference between species in the azimuthal profile or slope (Fig. 3g, h). Because spatial resolution is limited inversely by IO angle, maximum spatial resolution in both species is highest around 45° azimuth and 0° elevation, along the eye equator. This increase in equatorial spatial resolution might be an adaptation to terrain statistics of different habitats [33, 36], and due to the horizontal band of smaller ommatidial diameters at the eye equator formed during eye development [37, 38]. Regardless, this would improve the resolution of small objects near visual horizon, a feature that would help in avoiding predators and locating oviposition sites.

### Eye allometry in D. mauritiana RED3 prioritises contrast sensitivity more than D. simulans M3

In holometabolous insects, body size and the size of organs derived from imaginal discs depend on, and are proportional to, environmental factors like temperature and food availability during larval development [39, 40]. In flies, larval feeding has been shown to affect eye size, ommatidia size, and ommatidia count [41]. As a result, variation in eye size and composition may reflect rearing differences. To address this, we modelled the scaling relationships between eye surface area and the following measurements: total lens count, mean lens diameter, median IO angle, median equatorial (elevation = 0 ± 15°) IO angle, and IO angle interquartile range (Fig. 3i–m).

Eye surface area (*SA*) is an ideal reference for allometric scaling because it is proportional to the rate of light absorption of the entire eye. Also, because the ommatidial lenses almost completely cover the surface of the eye, the mean lens area (A) is approximately *SA* divided by the number of lenses (N), A ≈ *SA* / N, implying that *SA* ≈ N × A. This equation is approximate because we estimated A by assuming circular facets even though facet shapes vary. Because the number of discernible brightness levels is proportional to lens area, *SA* is also proportional to the total number of images the eye can resolve, its spatial information capacity [42]. Using ordinary least squares, we regressed each measurement on the sum of eye area and a dummy-coded species variable (Additional File 8: Table S2). We performed post hoc pairwise *t*-tests to compare means between species, defining the interspecific difference as *D. mauritiana* RED3–*D. simulans* M3, such that significant positive values mean that *D. mauritania* RED3 was greater than *D. simulans* M3 and vice versa for negative values. All models were a good fit, explaining a substantial proportion of the variance in *SA* plus the species variable (*R*^2^ = 0.65–0.97, *F* = 8–159, *P* ≤ 0.01).

Lens count and size had significant positive slope coefficients, such that larger flies have more and larger ommatidia in both species. However, lens count and lens diameter had significant interspecific differences after accounting for *SA*, but lens count was greater for *D. simulans* M3 and lens diameter was greater for *D. mauritiana* RED3. Therefore, *D. mauritiana* RED3 have lower ommatidial density than *D. simulans* M3. Conversely, the interquartile range (IQR) of lens diameters has a significant negative slope and a significant positive interspecies difference, implying that *D. mauritiana* RED3 lens diameters are more variable than *D. simulans* M3 after accounting for eye size. This is consistent with the lens volume eye maps discussed above, which found a greater range of lens sizes in *D. mauritiana* RED3 than *D. simulans* M3 along elevation, generally larger ommatidia for every azimuthal bin, and a greater azimuthal slope.

For both general and equatorial IO angles and comparison across both species, the slope coefficient was significant and negative, meaning that median angles scale inversely with eye size. However, the interspecific difference was only significant for median equatorial IO angles, such that *D. mauritiana* RED3 has significantly greater equatorial IO angles than *D. simulans* M3 after accounting for *SA*. Because spatial resolution is inversely proportional to IO angle, *D. simulans* M3 has greater spatial resolution at the eye equator but similar resolution elsewhere. The IQR of IO angles had an insignificant slope coefficient and a significant but negative interspecific difference, meaning that *D. simulans* M3 have a greater range of IO angles. This is consistent with the IO angle eye maps above, which found a greater range in the elevation profiles of IO angle in *D. simulans* M3 (Fig. 3f). The increase in IO angles near the boundaries of the eye should effectively increase the field of view (FOV) of the eye. Overall, these allometric relations suggest that *D. mauritiana* RED3 prioritise optical sensitivity more than *D. simulans* M3, which instead prioritise spatial resolution along the visual horizon and FOV at the peripheral extremes.

### D. mauritiana RED3 and D. simulans M3 optomotor responses trade off contrast sensitivity and spatiotemporal resolution

Our morphological analysis suggested that *D. simulans* M3 have higher spatial acuity due to smaller IO angles for equatorial ommatidia and *D. mauritiana* RED3 have higher optical sensitivity due to larger facet apertures, particularly in the central visual field just below the horizon. However, neural summation can recover sensitivity loss due to suboptimal optics by effectively sacrificing temporal or spatial resolution [41]. To measure the ethological implications of these optical differences, we used the flies’ optomotor response in a virtual reality flight simulator that allowed the presentation of different sinusoidal gratings moving to the left or right (Additional File 9: Fig. S7). Using a wingbeat analyser, we measured the flies’ steering effort in response to gratings of various contrasts, spatial frequencies, and temporal frequencies sorted randomly. Contrast sensitivity is defined here as the reciprocal of the lowest discernible contrast, and both spatial and temporal acuity are defined by the maximum discernible frequency. Assuming that the IO angle limits the maximal spatial sampling or resolution of the eye according to the Nyquist limit, such that the highest possible discernible frequency, *f*_*s*_, for a hexagonal lattice is given by the following equation: *f*_*s*_ = 1/√3 * *Δɸ*^−1^. So, for every *f*_*s*_, there is a corresponding ideal IO angle, *Δɸ* = 1/√3 * *f*_*s*_^−1^.

**Fig. 4:**
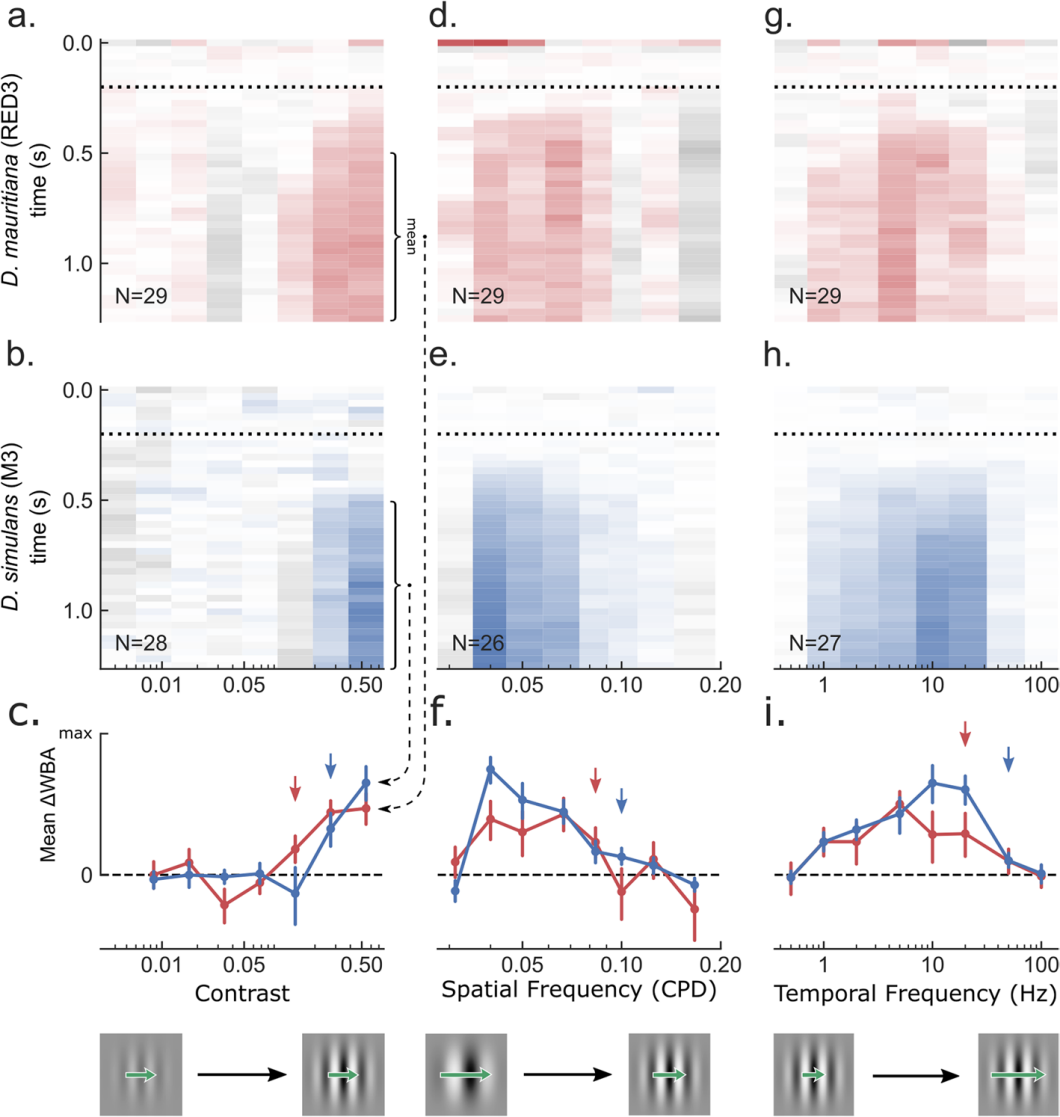
Behavioural measurement of *D. simulans* and *D. mauritiana* contrast sensitivity, spatial resolution, and temporal resolution. Gratings of various contrasts (**a**–**c**), spatial frequencies (**d**–**f**), and temporal frequencies (**g**–**i**) were presented to 3 males and 3 females from each of the two species in a rigid tether flight simulator equipped with a wingbeat analyser. The gratings were filtered through a Gaussian window and remained still for .2 s before moving to the left or right, indicated by the dotted line. For each subject, responses to leftward moving gratings were averaged with responses to the same grating moving rightward so that positive values represent mean steering in the direction of the grating (red or blue) and negative represents counter steering (grey). Mean normalised responses taken between .5 and 1.25 s were baseline corrected, subtracting the mean response during the .1 s before the onset of motion. Two of these ranges are indicated by annotations in **a** and **b**. connected by dashed arrows to their mean in **c**. Sample sizes are indicated in the bottom left corner of the colourmaps. The images of gratings in the bottom of **c**, **f**, and **i** are meant to give a sense of the change in the stimulus along the *x*-axis. Green arrows indicate the change in speed of the grating, f_t_/f_s_, which remains constant in the contrast experiment, decreases in the spatial frequency experiment, and increases in the temporal frequency one. **a**–**c** As contrast increases, RED3 begins responding significantly at .14 (red arrow in **c**) and M3 at .27 (blue arrow in **c**). **d**–**f** As spatial frequency increases and therefore rotational velocity decreases, mean responses decrease gradually until the Nyquist limit determined theoretically by the IO angle, reducing the contrast for higher frequencies as a result of aliasing. This limit differed between the two species, with RED3 responding significantly to spatial frequencies as high as .08 CPD (red arrow in **f**) and M3 as high as .1 CPD (blue arrow in **f**). **g**–**i** As temporal frequency and therefore rotational velocity increases, mean responses increase until they reach the Nyquist limit determined by the temporal resolution of the optomotor response, reducing the contrast for higher frequencies. M3 demonstrated higher temporal acuity, responding significantly to frequencies as high as 50 Hz (blue arrow) while RED3 stopped at 20 Hz (blue arrow). Data provided in Fig. 4_share.zip on figshare [29]

In the flight arena, *D. simulans* M3 and *D. mauritiana* RED3 traded off between higher contrast sensitivity and spatiotemporal tuning (Fig. 4). In accord with their larger ommatidia, *D. mauritiana* RED3 demonstrated higher contrast sensitivity (0.14^−1^ = 7.4) than M3 (0.27^−1^ = 3.7). Conversely, the spatial tuning curves demonstrate that *D. simulans* M3 has a higher spatial acuity (0.1 cpd) than *D. mauritiana* RED3 (0.08 cpd), implying smaller IO angles (~ 5.8° versus ~ 7.2°) consistent with smaller measured IO angles in the eye equator of *D. simulans* M3. *D. simulans* M3 also responded with greater strength around 0.04 cpd, likely supported by their wider peripheral IO angles and greater IQR. Lastly, *D. simulans* M3 demonstrated higher temporal acuity, 50 Hz, than *D. mauritiana* RED3, 20 Hz. Overall, this demonstrates sharper (higher spatial acuity) and faster vision (higher temporal acuity) in *D. simulans* M3 but a greater ability to compare brightness values (higher contrast sensitivity) in *D. mauritiana* RED3.

## Discussion

The evolution of variation in overall eye size, ommatidia number and facet area has been shown within and between *Drosophila* species by several research groups and differences in vision have been proposed based on these optical parameters [16–21]. *D. mauritiana* has evolved generally larger eyes composed of wider ommatidia than its sibling species *D. simulans* and *D. sechellia* since their divergence approximately 240,000 years ago [16, 17, 21, 26, 43, 44]. The eye size of their common ancestor was likely similar to *D. simulans* because their sister species *D. melanogaster* has more similar sized eyes to this species rather than *D. mauritiana* [21]. In this study, the demonstrated allometries and regional specialisations of *D. mauritiana* and *D. simulans* were found to differ in quantity but not quality: (1) maximum sensitivity in the central visual field below the horizon, with very similar elevation and azimuthal profiles; (2) maximum acuity along the visual horizon of the eye; and (3) improvements in optical sensitivity and spatial resolution for larger conspecifics. Future investigations of the developmental origins of these gradients and regional specialisations in spatial resolution and optical sensitivity and how they may differ between flies will aid our understanding of how the astonishing diversity in insect eyes has evolved.

So far, our knowledge of how insects developmentally and evolutionarily balance the trade-off between ommatidia number (resolution) and ommatidia size (sensitivity) is very limited. The genetic basis of evolutionary differences in eye size has been difficult to determine, partly because ommatidia size and number seem to be genetically uncoupled and differences in these features polygenic. While differences in ommatidia size between *D. mauritiana* and *D. simulans* have been mapped to *orthodenticle* [17, 26], and a cis-regulatory region of *eyeless* has been shown to contribute to differences in eye size within *D. melanogaster* and between this species and *D. pseudoobscura* [20], these changes do not explain the full extent of variation. Other genes involved in the regulation of cell proliferation and differentiation in developing eye imaginal discs are the most likely candidates to contribute to the diversification of eye size. For example, phosphoinositides including the *Drosophila* class I(A) PI 3-kinase Dp110 and its adaptor p60, the gap-junction protein *inx2* and the 40 s ribosomal protein S6 kinases, have all been shown to alter ommatidia number and/or size [45–48].

Very little comparative functional data is available to truly understand the impact of natural variation in eye structure on vision. Here we modelled and tested optical capacity in two *Drosophila* species—*D. mauritiana* and *D. simulans*—between two strains that had similar ommatidia number but significantly different ommatidia facet sizes, to assess whether predicted differences in contrast sensitivity, spatial resolution, and temporal resolution could be observed in behavioural experiments. In principle, larger facets could evolve to provide either better sensitivity (collecting more light in a similar amount of time) or better temporal resolution (collecting similar amounts of light over shorter times), or some combination. Indeed, we confirmed higher spatial and temporal acuity in *D. simulans* with smaller ommatidia and improved contrast sensitivity in M3, with larger ommatidia. Whether and how these differences reflect meaningful adaptations to ecological differences remains to be explored, but the recapitulation of morphological divergence through behavioural paradigms is compelling.

We also identified substantial intraocular variation in lens volume, interommatidial spherical angles, facet shape, and lens diameter, as has been reported in other dipterans. While the optomotor experiments reported here tested global responses, future behavioural experiments might target different parts of the visual field to see whether this regional variation has a functional significance. Potentially increased spatial resolution at the equator of the eye, and in the anterior-ventral FOV, was predicted from our morphological data. Increased acuity at the horizon, combined with horizontally narrower facets at the centre of the eye, for example might enhance the detection of lateral optic flow. Likewise, the stronger anterior frontal gradient in predicted resolution for females could have implications for the detection of oviposition sites.

While our analysis supports the use of 3D morphological data to predict optical capacity, many other factors are involved in information acquisition and processing in the insect eye that are not as easily accessible. Recent discovery of smooth and saccadic retinal muscle movement to improve perception of moving and stationary objects respectively [49, 50] as well as hyperacute vision via photomechanical photoreceptor contractions (microsaccades) [51–54] have revealed much more sophisticated mechanisms are employed in *Drosophila* eyes to sample visual information. In particular, the spatial resolution of compound eyes can exceed the spatial Nyquist limit set by the IO angle due to brief, stereotyped photomechanical contractions (microsaccades) that sharpen and shift rhabdomere receptive fields, affording so-called hyperacuity [52]. These contractions are optimal for processing brief bursts of light followed by periods of darkness to better match the refractory phase of rhabdomere microvilli [52] and generally match the optical flow of forward translation [54]. As a result, these phases of improved acuity apply to specific combinations of motion direction, duration, speed, and visual field region. Moreover, the advantages and magnitude of the photomechanical rhabdomere contractions are limited by IO angle [53], so that the difference in IO angles measured here still confer an important difference in visual capacity.

The complexity of the visual system overall, incorporating mechanisms of neural summation and hyperacuity, further highlights the importance of using behavioural measurements of acuity and sensitivity and reinforces the conceptual distinction between optical and contrast sensitivity. Neural summation could have reversed these differences as it did for *D. mojavensis* due to darkness adaptation [55] or facultatively within *D. melanogaster* individuals in response to forward optical flow [56]. An assessment of the optics alone would have ignored the difference in temporal acuity and overestimated the difference in contrast sensitivity between *D. mauritiana* and *D. simulans* based on differences in optical sensitivity*.*

Aside from the functional aspect, the maintenance of eyes and the underlying complex neurocircuits are a metabolically expensive investment [57]. For example, comparison between photoreceptor information rates of larger and more active flies like the blowfly *Calliphora* with the smaller *D. melanogaster* showed a five times higher performance in *Calliphora* but at a ten times higher energetic cost [58]. The evolution of overall larger eyes with more and wider ommatidia and resulting increase in contrast sensitivity in *D. mauritiana* must therefore represent an economically viable investment aligned to their specific optical needs. The balance between sensory system requirements and energy efficiency has been observed in other fly species: The male housefly (*Musca domestica*) has a 60% higher bandwidth (measure of speed of response) in their contrast-coding R1-6 compared to females, allowing them to track these females in flight, whereas bandwidth decreases in male blowflies by 20% towards the back of the retina [59, 60]. It is therefore conceivable that absolute eye size is under stronger selection than ommatidia number or ommatidia size on their own, and at least to some extent independent of body size and other functional traits [61–63]. Evidence from *Drosophila* wings suggests that compensatory mechanisms guarantee a certain wing size if overall size deviates too much [64, 65]. A similar mechanism could be at play in *D. simulans* where ommatidia number and size seem to be coordinated to maintain similar eye size across strains.

## Conclusions

Insects play vital roles in various ecosystems, including pollination and decomposition. Climate change and the disappearance of ecological niches around the world highlights the need to understand how they perceive and interact with their environment and vision is a primary sensory modality for many insects, shaping their behaviour, foraging strategies, and reproductive patterns. Our study demonstrates that even subtle differences in ommatidia size between closely related species can have a measurable effect on their vision. Therefore, comparative studies of natural variation in eye morphology and the consequences for vision across dipterans and beyond are needed to fully understand how the diversification of eye size, shape, and function allowed insects to adapt to the vast range of ecological niches around the world.

## Methods

### Fly strains and husbandry

Multiple strains of *D. simulans* and *D. mauritiana* were used in this study [21] (Additional File 1: Table S1). All stocks used were kept on standard yeast extract-sucrose medium at 25 °C under a 12:12-h dark/light cycle. For experiments, flies were reared at controlled, low density, achieved by transferring set numbers of males and females (typically between 10 and 20 of each sex) into fresh food containers to lay offspring. Adult offspring were removed soon after eclosion for experiments.

### Scanning electron microscopy

Fly heads were prepared and imaged as previously described [21]. Briefly, heads were fixed in Bouin’s solution (Sigma-Aldrich) and dehydrated to 100% ethanol. For SEM imaging heads were critical point dried in a Tousimis 931.GL Critical Point Dryer and mounted onto sticky carbon tabs on SEM stubs, gold coated (10 nm) and imaged in a Hitachi S-3400N SEM with secondary electrons at 5 kV.

### Morphological measurements

SEM images of eyes were analysed using FIJI/ImageJ [66]. For each strain, 15 males and 15 females (except MS17 females: *n* = 11) were analysed. Ommatidia number was counted manually by using multi-point tool for one compound eye per individual (from side views of compound eyes). Ommatidia size and overall eye area were measured manually with the polygon selection tool. Frontal and central ommatidia area were measured for each eye with the polygon selection tool. The area of six central ommatidia was average to determine mean ommatidia (facet) size. Wing and tibia of the second leg of each fly were dissected in 70% ethanol and mounted in Hoyer’s solution and cured overnight at 60 °C. Wings and tibia were imaged at × 5 (× 1.25) magnification using a Zeiss Axioplan microscope equipped with a ProgRes MF cool camera (Jenaoptik). Wing and tibia size were measured using the line tool in Fiji/ImageJ. Plots and statistical analysis were carried out in RStudio Version 2023.03.0 + 386 using the Tidyverse suite of packages [67]. Where analysis required comparison between a length and an area, the length measurement was squared. Linear lines of fit were added to plots using geom_line(stat = "smooth", method = lm). For correlation analysis, data was first checked for normality using Shapiro–Wilk and then tested using either Pearson’s correlation coefficient or Spearman’s rank correlation coefficient using stat_cor(method = "pearson") or stat_cor(method = "spearman"). Standard deviations for the raw measurements were calculated in Excel.

### Synchrotron radiation tomography

Fly heads were prepared as described for SEM to 100% ethanol, then stained with 1% iodine and washed in ethanol. Fly heads were mounted in 20-µl pipette tips filled with 100% ethanol for synchrotron radiation X-ray tomography and scanned at the TOMCAT beamline of the Swiss Light Source (Paul Scherrer Institute, Switzerland [68] and Diamond-Manchester Imaging Branchline I13-2 (Diamond Light Source, UK) [69, 70] as previously described [21, 26].

### 3D segmentation

The IMOD Software package [71] was used to generate cropped mrc stacks for 3D segmentation and analysis of the head tissue, lenses, and optic lobes in Amira v.2019.2 (Thermo Fisher Scientific). Ommatidial lenses were segmented through threshold and separate objects tools. Lens sizes were analysed and colour-coded depending on size with the label analysis and sieve module.

### Morphometric analysis

Heads were tilted in the SEM to obtain flat images of frontal and central ommatidia for geometric morphometric analysis. The six corners of each facet were landmarked using the *digitize2D* function in the R package Geomorph (v.4.0.4) [72, 73]. Data were registered and Procrustes transformed using *procSym* function in the package Morpho (v.2.10) [74] to account for reflection, before principal component analysis using the Geomorph package. Hierarchical clustering was performed and visualised using the factomineR (v.2.6) and factoextra (v.1.0.7) [75, 76] packages, using the option *nb.clusters* = *-1* to select the optimal number of clusters. The strain, sex, and positional identities of the resulting clusters were analysed by chi-square in base R, and the contribution of the principal components to clustering was extracted from *desc.var* generated by the *HCPC* function for clustering. Plots were generated using ggplot2 (v.3.4.2) [77] *n* = 30.

### ODA and allometry

To approximate the optical performance of the two species, we processed CT stacks of six flies (three males and three females) from the RED3 strain of *D. mauritiana* and the M3 strain of *D. simulans.* This allowed us to apply the 3D ommatidia detecting algorithm (ODA-3D; Additional File 9: Figure Fig. S7a), a pipeline for automatically measuring a number of visual parameters for compound eyes [33]. Each dataset was manually cleaned to generate binary images of only the corneal lenses. Then, the programme fitted a cross-sectional surface through the coordinates of the lens cluster and projected these coordinates onto the cross-section, allowing a custom clustering algorithm to find ommatidia-like objects in the 2D projected images. Finally, the volume, diameter, cross-sectional area, length, and average IO angle of each lens were measured. Eye surface area was estimated as the sum of the lens areas based on the ODA-derived lens diameters. Allometric scaling relations were derived by regressing each of the measured visual parameters on eye surface area plus a dummy-coded species variable. Post hoc pairwise *t*-tests were used to compare means between species. The interspecies difference was defined as *D. mauritiana* RED3–*D. simulans* M3, such that a significant positive difference implies that *D. mauritiana* RED3 was greater than *D. simulans* M3 after effectively accounting for differences in eye surface area (*SA*). The resultant parameters of these models are found in Additional File 8: Table S2.

### Flight arena

To measure the optomotor performance of *D. mauritiana* RED3 and *D. simulans* M3, we performed psychophysics in a rigid tether flight simulator equipped with a wingbeat analyser (Additional File 9: Fig. S7b). Flies from 3 to 6 days post-eclosion were cold-anesthetised for about 5 to 30 min, glued to a 2-mm-diameter tungsten rod, and left to recover for about an hour with a piece of paper placed on their feet to prevent wing beating. They were then centred within an acrylic cube lined with rear-projection material (with 1/6 of the panels left open), immersing them in the projection surrounding 5/6 of their FOV (Additional File 9: Fig. S7c). Stimuli were generated by a computer using a custom open-source graphics library and projected onto the front panel of the arena at 120 Hz by a high-speed projector (technical information can be found in Currea, Smith and Theobald [41] and Currea et al. [55]. An IR light cast the shadow of each wing onto photodiodes below the fly designed to output the amplitude of each wingbeat shadow as a 1000-Hz voltage signal. The difference between the left and right wingbeat amplitudes (ΔWBA) is proportional to yaw torque and indicates the fly’s steering effort. For instance, in Additional File 9: Fig. S7d, we plot the ΔWBA time series for an exemplary fly in response to 9 gratings of different contrast (corresponding to the line’s saturation) moving to the left or right (warm vs. cool hue). Note that the strength of the response is affected by contrast while the direction corresponds generally to the direction of motion. These responses were taken from Currea et al. [55], which used the same methods.

### Psychophysics

In the flight arena, flies viewed sinusoidal moving gratings of various Michelson contrasts (0, 0.01, 0.02, 0.04, 0.08, 0.16, 0.32, 0.64; Fig. 4a–c, with examples at the bottom of c), spatial frequencies (1.2, 1.4, 1.8, 2.3, 2.9, 3.8, 4.8, 5.7, 7.2, 9.6 cycles/°; Fig. 4d–f), and temporal frequencies (0.1, 0.2, 0.5, 1, 2, 5, 10, 20, 50, 100 Hz; Fig. 4g–i) to measure the functional consequences of their optical differences. The gratings were filtered through a Gaussian window and remained still for 0.2 s before moving to the left or right, indicated by the dotted lines in Fig. 4. For each subject, responses to leftward moving gratings were (1) averaged with responses to the same grating moving rightward, (2) baseline corrected, subtracting the mean response during the 0.1 s before the onset of motion, and (3) normalised to the maximum mean response per fly so that positive values represent mean steering in the direction of the grating, with a maximum of 1 (fully saturated red or blue) and negative represents countersteering (grey). These baseline-corrected normalised responses were averaged across each group to make the colormaps in Fig. 4. For each fly, an average of these normalised responses was taken from 0.5 to 1.25 s and used for plotting and comparing means in the bottom row of subplots in Fig. 4.

Bootstrapping was used to test for a grating’s discernibility by estimating the standard error of the mean and 90% C.I.s for the mean response. We bootstrapped the means taken between 0.5 and 1.25 s 10,000 times at the subject level to generate empirical sampling distributions of the mean for each parameter value accounting for repeated measures. The 68% C.I. of each distribution was used as an approximation of the standard error (error bars in the bottom row of Fig. 4) and the lower bound of the 90% C.I. was used to test for positive significance with a two-tailed alpha of 0.1 or one-tailed alpha of 0.05. Contrast sensitivity was defined as the reciprocal of the lowest discernible contrast and spatial and temporal acuity were defined by the highest discernible frequency.

### Supplementary Information

**Additional file 1: Table S1.** *Drosophila* natural strains used in this publication.

**Additional file 2: Fig. S1.** Correlation analysis for ommatidia number and ommatidia size *D. simulans* and *D. mauritiana* strains. Male and female *D. simulans* (blue) show a significant, negative correlation between ommatidia size and ommatidia number such that individuals with larger ommatidia tend to have less ommatidia overall. In *D. mauritiana* (red), males exhibit a significant positive correlation between ommatidia size and number, where the individuals with larger ommatidia also have a larger number of ommatidia. n (females) = 146, (males) = 150. Raw measurements provided in Fig. 1 morphological measurements.xlsx on figshare [29].

**Additional file 3: Fig. S2.** Variation in wing and tibia size across *D. mauritiana* and *D. simulans* strains. Average eye size (circle area) of *D. simulans* (blue) and *D. mauritiana* (red) strains (circle labels) is plotted against wing vein and tibia lengths. *D. simulans* strains (blue) generally have larger wings but show some variation in tibia size whereas *D. mauritiana* strains generally have smaller wings and greater variation in tibia length. *n* = 11 for MS17 females and *n* = 15 for males and females of all other strains. Raw measurements provided in Fig. 1 morphological measurements.xlsx on figshare [29].

**Additional file 4: Fig. S3.** Correlation of 2nd leg tibia size with eye size in *D. mauritiana* and *D. simulans* strains. In males and females of both species only a subset of strains shows a significant positive correlation between tibia length and eye size. *n* = 11 for MS17 females and *n* = 15 for males and females of all other strains. Raw measurements provided in Fig. 1 morphological measurements.xlsx on figshare [29].

**Additional file 5: Fig. S4.** Correlation of wing size with eye size in *D. mauritiana* and *D. simulans* strains. In males and females of both species only a subset of strains shows a significant positive correlation between wing vein length (a proxy for wing size) and eye size. *n* = 11 for MS17 females and *n* = 15 for males and females of all other strains. Raw measurements provided in Fig. 1 morphological measurements.xlsx on figshare [29].

**Additional file 6: Fig. S5.** Eye maps of ommatidia measurements across eyes of *D. simulans* and *D. mauritiana.* Eye maps of ommatidial lens volumes (a.), lens diameters (b.), and cross-sectional areas (c.), with their elevation (right column of each panel) and azimuthal (bottom row of each) profiles, and their azimuthal slope (bottom right inset of each) from 6 flies from each of the two species, *D. mauritiana* (RED 3) and *D. simulans* (M3), 3 males and females for each. The eyes are sorted in order from smallest to largest eye surface area, which also resulted in ordering by sex because males are generally smaller. Each dot of the scatter plot represents the location of an individual ommatidium in polar coordinates coloured by its 3D volume according to the colour bars. Line colours in the azimuthal and elevation profiles and dot colours in the azimuthal slope plots indicate the fly’s rank in order of eye size per species, such that the darkest one is the largest eye of that species. Each outcome is divided into 20 evenly spaced bins of elevation (line plots to the right) and azimuth (line plots below) with error bars indicating 3 times the standard error of the mean of each bin. Ordinary least squares was used to regress each outcome on azimuthal position to estimate and compare the azimuthal slope between the two species. Scatterplots in the bottom right show the resulting slope coefficients from those models. Note that azimuth here is in radians but was converted to degrees for the plots Fig. 3 and the calculation of the slope. a. Note that this presents the full dataset used in the elevation profiles of Fig. 3b and the azimuthal slopes in Fig. 3d. Data provided in Fig. 3_share.zip on figshare [29].

**Additional file 7: Fig. S6.** Eye maps of ommatidia lens length and IO across eyes of *D. simulans* and *D. mauritiana. a–b.* Eye maps of ommatidial lens length (a.) and IO angle (b.) as in Suppl. Figure 5, with their elevation (right column of each panel) and azimuthal (bottom row of each) profiles, and their azimuthal slope (bottom right inset of each) from 6 flies from each of the two species, *D. mauritiana* (RED 3) and *D. simulans* (M3), 3 males and females for each. The eyes are sorted in order from smallest to largest eye surface area, which also resulted in ordering by sex because males are generally smaller. Each dot of the scatter plot represents the location of an individual ommatidium in polar coordinates coloured by its 3D volume according to the colour bars. Line colours in the azimuthal and elevation profiles and dot colours in the azimuthal slope plots indicate the fly’s rank in order of eye size per species, such that the darkest one is the largest eye of that species. Each outcome is divided into 20 evenly spaced bins of elevation (line plots to the right) and azimuth (line plots below) with error bars indicating 3 times the standard error of the mean of each bin. Ordinary least squares was used to regress each outcome on azimuthal position to estimate and compare the azimuthal slope between the two species. Scatterplots in the bottom right show the resulting slope coefficients from those models. Note that azimuth here is in radians but was converted to degrees for the plots Fig. 3 and the calculation of the slope. b. Note that, as in Fig. 3, the elevation profile for IO angle was plotted differently because plotting the binned averages obfuscates the horizontal band of high acuity along the equator, likely due to the large range of IO angles along azimuth. This also presents the full dataset used in Fig. 3f and the azimuthal slopes in Fig. 3h. Data provided in Fig. 3_share.zip on figshare [29].

**Additional file 8: Table S2.** Parameters of the linear regression models of the allometries of optical parameters with respect to eye surface area. Each outcome was modelled as a linear combination of eye area and species (dummy-coded) with a constant intercept using ordinary least squares regression. The coefficient of determination (R^2^) and F-statistic are provided as measurements of goodness-of-fit with asterisks indicating the significance according to the key at the bottom of the table. The intercept and slope are the resulting coefficients of the regression model. *D. mauritiana* RED3 – *D. simulans* M3 is the pairwise difference of means after accounting for differences in eye size, such that values < 0 imply that *D. simulans* M3 values were greater than *D. mauritiana* RED3 relative to eye size. The significance of these statistics (F, intercept, slope, and *D. mauritiana* RED3 – *D. simulans* M3) is signified by the number of asterisks next to these values according to the key at the bottom of the table.

**Additional file 9: Fig. S7.** Workflow for modelling and testing vision in *Drosophila*. a. Optical performance was evaluated across the visual field of each eye by applying the ODA-3D, which requires first prefiltering the stack to just its corneal lenses to fit a cross-sectional surface, apply a clustering algorithm, and finally take optically relevant measurements for each lens. b. Optomotor performance was evaluated by a virtual reality flight simulator using an open-source computer graphics library, high-speed projector, and precisely positioned first-surface mirrors to project high resolution and contrast stimuli surrounding 5/6 of the flies’ FOV. c. Flies were glued to a thin tungsten rod and centred within an acrylic cube lined with rear-projection material immersing them in the projection as in b. An IR light casted the shadow of each wing onto photodiodes below the fly designed to output the amplitude of each wingbeat shadow as a 1000 Hz voltage signal. The difference between the left and right wingbeat amplitudes (ΔWBA) is proportional to yaw torque and indicates the fly’s steering effort. d. We plotted the ΔWBA time series for an exemplary fly in response to 9 gratings of different contrast (corresponding to the line’s saturation) moving to the left or right (warm vs. cool hue), drawn from(Currea et al*.*, 2022). Notice that the strength of the response is partially dependent on contrast while the direction corresponds generally to the direction of motion. Leftward motion ΔWBA responses were averaged with the inverse of rightward motion ΔWBA responses to account for directional biases in our measurement. These averages were then normalised to the maximum mean response per fly and averaged across each group to make the colormaps in Fig. 4. For each fly, an average of these normalised responses was taken from 0.5–1.25 s and used for plotting and comparing means in the bottom subplots (Fig. 4 c, f, and i). e. Sinusoidal moving gratings were used because they are independently defined by a single orientation (leftward or rightward, for example), spatial frequency (x-axis), contrast (y-axis), and temporal frequency (the frequency of brightness change per pixel).

## Data Availability

All data generated or analysed during this study are included in this published article, its supplementary information files and publicly available repositories. All data and analysis scripts for the ODA-3D results (Fig. 3), flight arena optomotor results (Fig. 4), and morphological measurements (Fig. 1) are available in a publicly available figshare repository, [29]. 10.6084/m9.figshare.24769677. Raw data of synchrotron scans analysed in this study are available from the corresponding author on request.

## Abbreviations

IO: Interommatidial angles
Otd: Orthodenticle
IQR: Interquartile range
FOV: Field of view
