# Supplementary material for: Evolution of compound eye morphology underlies differences in vision between closely related Drosophila species
Source: BMC Biol. 2024 Mar 19;22:67. doi: 10.1186/s12915-024-01864-7 (PMC10953123; doi:10.1186/s12915-024-01864-7)
Supplement: Supplementary file 1 — Additional file 1: Table S1. Drosophila natural strains used in this publication. [file 12915_2024_1864_MOESM1_ESM.pdf]

Table S1 - *Drosophila* strains used in this eye size survey.

Line	Species	Collection place	Collector/ Provider Lab	Collection date	Reference
M3	*D. simulans*	Antananarivo, Madagascar	B. Ballard/ Christian Schlötterer	1998	Palmieri et al. 2014 MolEc Res
DAV23	*D. simulans*	Davis, California, USA	Michael Turelli	2009	
M252	*D. simulans*	Antananarivo, Madagascar	B. Ballard/ Christian Schlötterer	1998	Palmieri et al. 2014 MolEc Res
HIN10	*D. simulans*	Entawville, South Carolina, USA	Paul Schmidt/ Christian Schlötterer	2008	
Kib11	*D. simulans*	Kibale, Uganda	Marianne Imhof/ Christian Schlötterer	2001	Nolte and Schlotterer Genetics 2008, Hilbrant et al. 2014 BMC Evol. Biol.
MAV2	*D. mauritiana*	Mareaux Vacoas, Mauritius	D. Nunes/ Christian Schlötterer	2009	
RED3	*D. mauritiana*	Reduit, Mauritius	M. Ramos/ Christian Schlötterer	2006	
MS17	*D. mauritiana*	Seizieme Mille, Mauritius	Kyoto DGGR, stock number E-18912	1987	Nolte V. et al. 2013 Genome Res.
TAM16	*D. mauritiana*	Tamarin, Mauritius	Christian Schlötterer/ Christian Schlötterer	2007	Nolte V. et al. 2013 Genome Res.
