# Supplementary figures and images for: Evolution of compound eye morphology underlies differences in vision between closely related Drosophila species
Source: BMC Biol. 2024 Mar 19;22:67. doi: 10.1186/s12915-024-01864-7 (PMC10953123; doi:10.1186/s12915-024-01864-7)

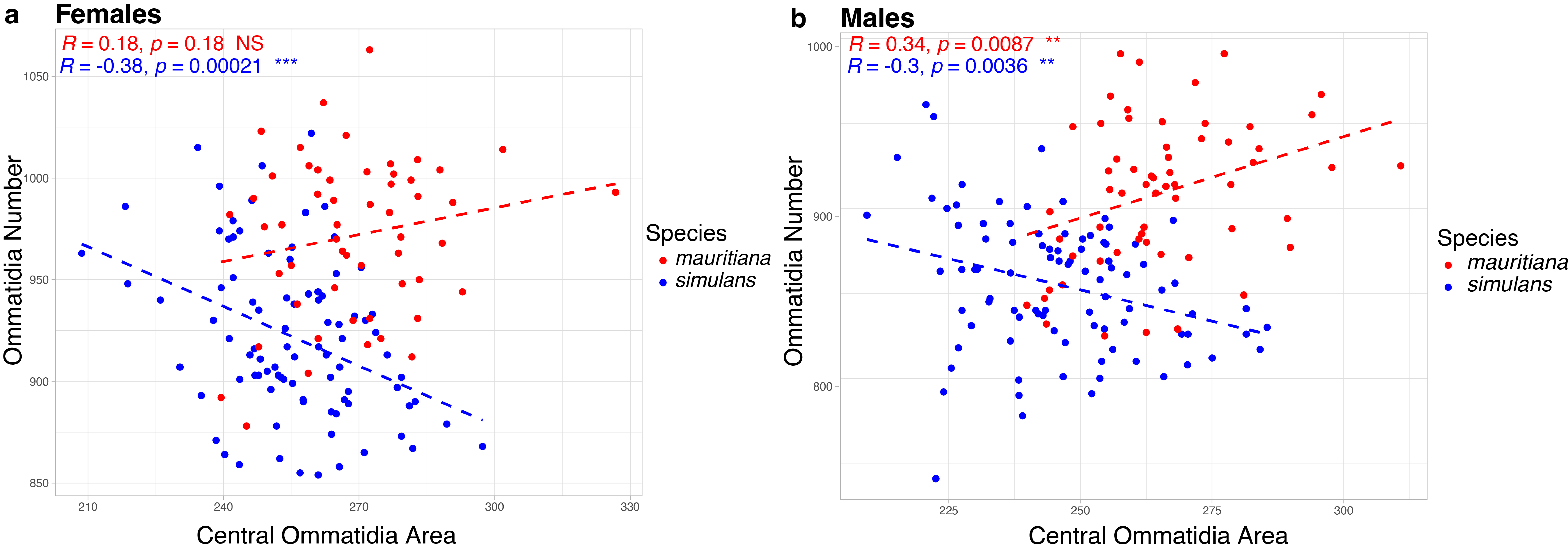

Supplement: Supplementary file 2 — Additional file 2: Fig. S1. Correlation analysis for ommatidia number and ommatidia size D. simulans and D. mauritiana strains. Male and female D. simulans (blue) show a significant, negative correlation between ommatidia size and ommatidia number such that individuals with larger ommatidia tend to have less ommatidia overall. In D. mauritiana (red), males exhibit a significant positive correlation between ommatidia size and number, where the individuals with larger ommatidia also have a larger number of ommatidia. n (females) = 146, (males) = 150. Raw measurements provided in Fig. 1 morphological measurements.xlsx on figshare [29]. [file 12915_2024_1864_MOESM2_ESM.png]

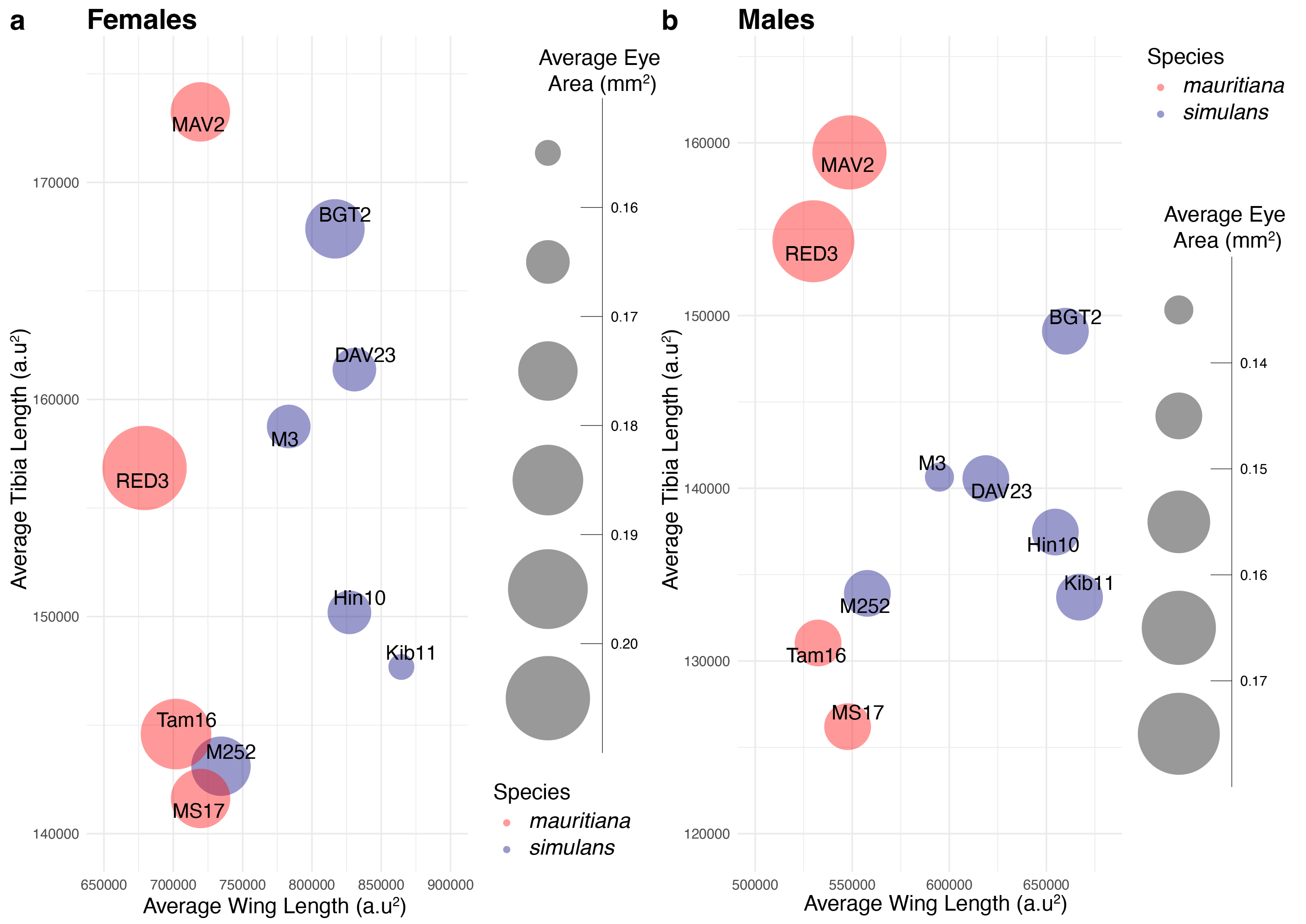

Supplement: Supplementary file 3 — Additional file 3: Fig. S2. Variation in wing and tibia size across D. mauritiana and D. simulans strains. Average eye size (circle area) of D. simulans (blue) and D. mauritiana (red) strains (circle labels) is plotted against wing vein and tibia lengths. D. simulans strains (blue) generally have larger wings but show some variation in tibia size whereas D. mauritiana strains generally have smaller wings and greater variation in tibia length. n = 11 for MS17 females and n = 15 for males and females of all other strains. Raw measurements provided in Fig. 1 morphological measurements.xlsx on figshare [29]. [file 12915_2024_1864_MOESM3_ESM.png]

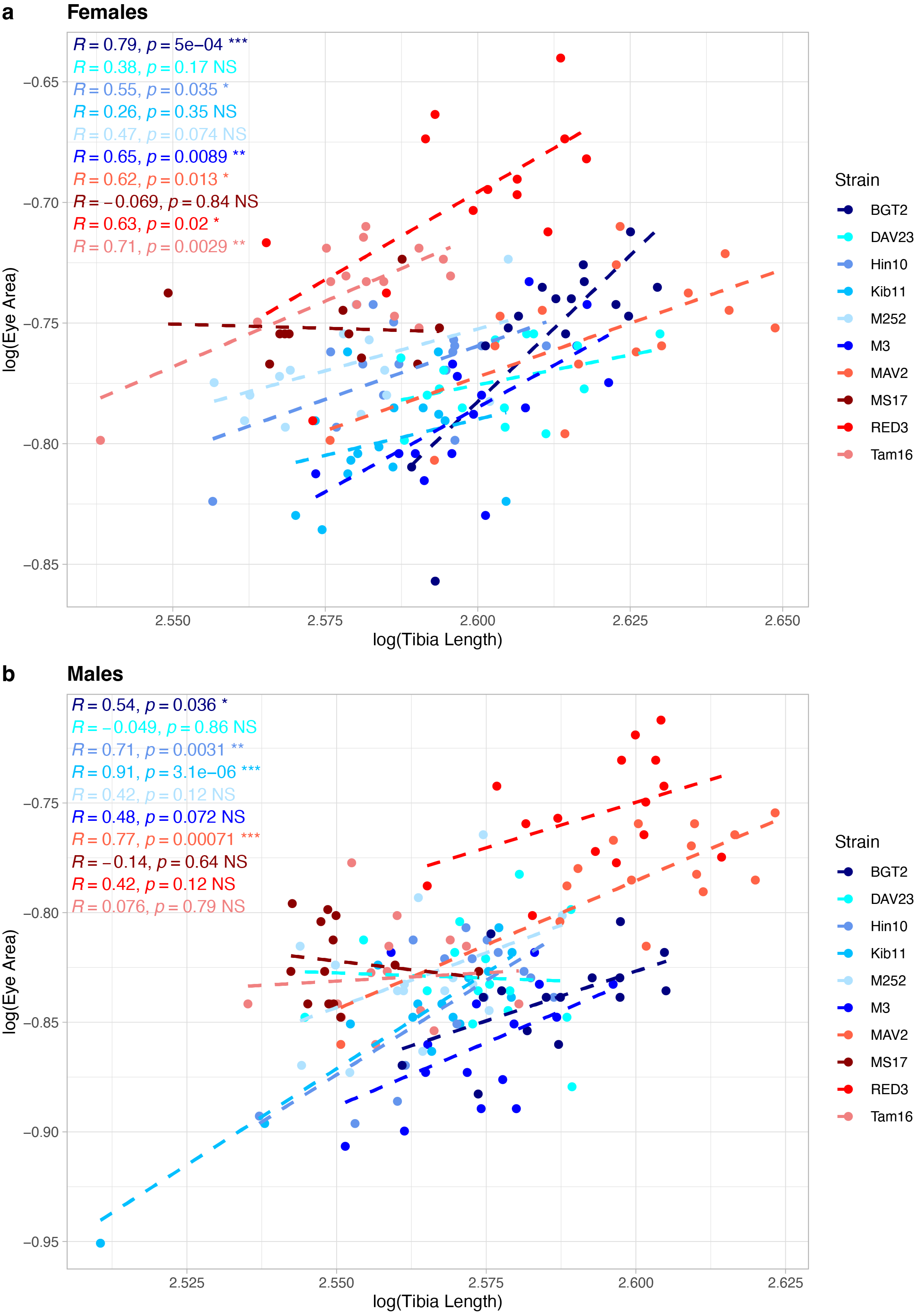

Supplement: Supplementary file 4 — Additional file 4: Fig. S3. Correlation of 2nd leg tibia size with eye size in D. mauritiana and D. simulans strains. In males and females of both species only a subset of strains shows a significant positive correlation between tibia length and eye size. n = 11 for MS17 females and n = 15 for males and females of all other strains. Raw measurements provided in Fig. 1 morphological measurements.xlsx on figshare [29]. [file 12915_2024_1864_MOESM4_ESM.png]

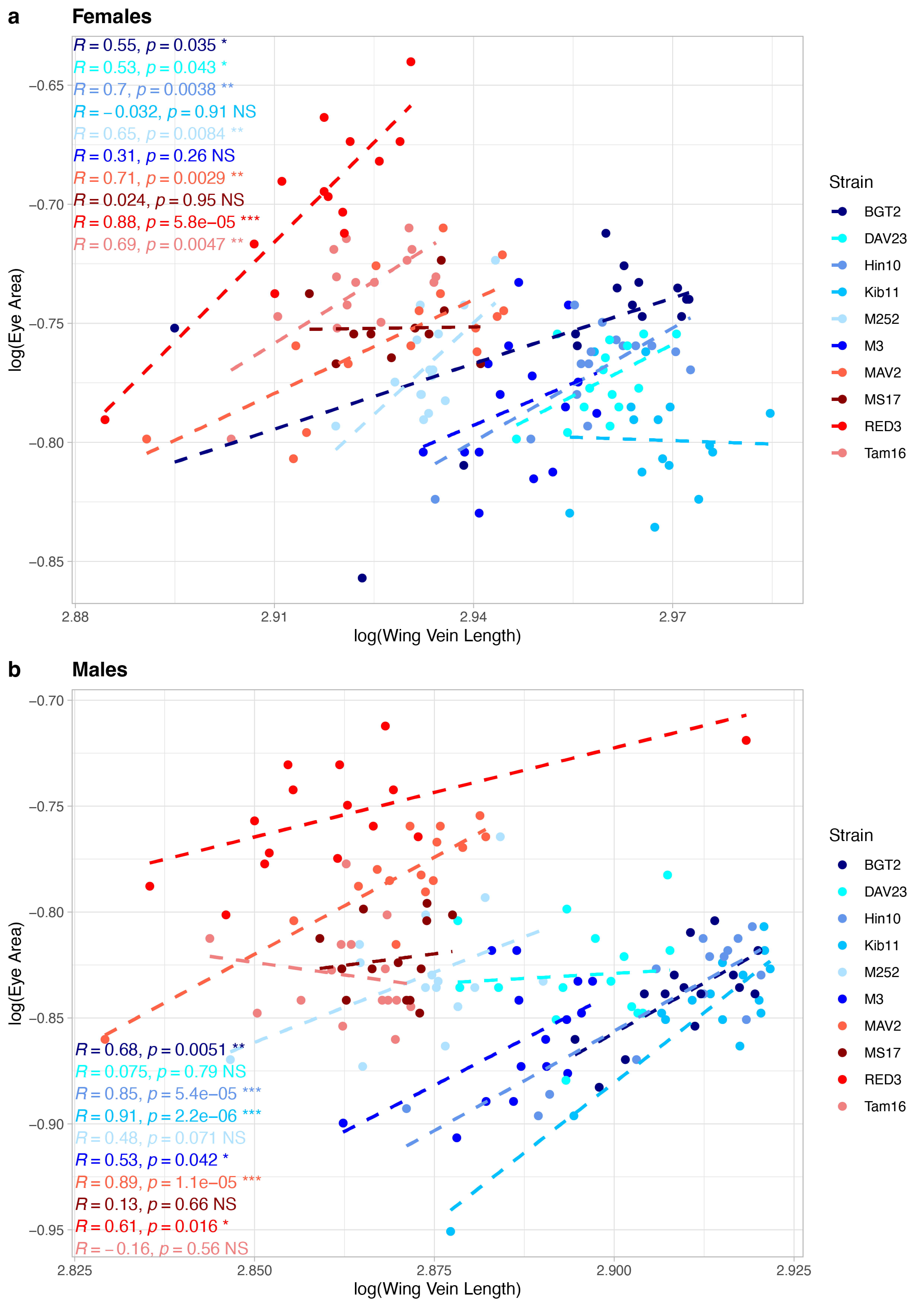

Supplement: Supplementary file 5 — Additional file 5: Fig. S4. Correlation of wing size with eye size in D. mauritiana and D. simulans strains. In males and females of both species only a subset of strains shows a significant positive correlation between wing vein length (a proxy for wing size) and eye size. n = 11 for MS17 females and n = 15 for males and females of all other strains. Raw measurements provided in Fig. 1 morphological measurements.xlsx on figshare [29]. [file 12915_2024_1864_MOESM5_ESM.png]

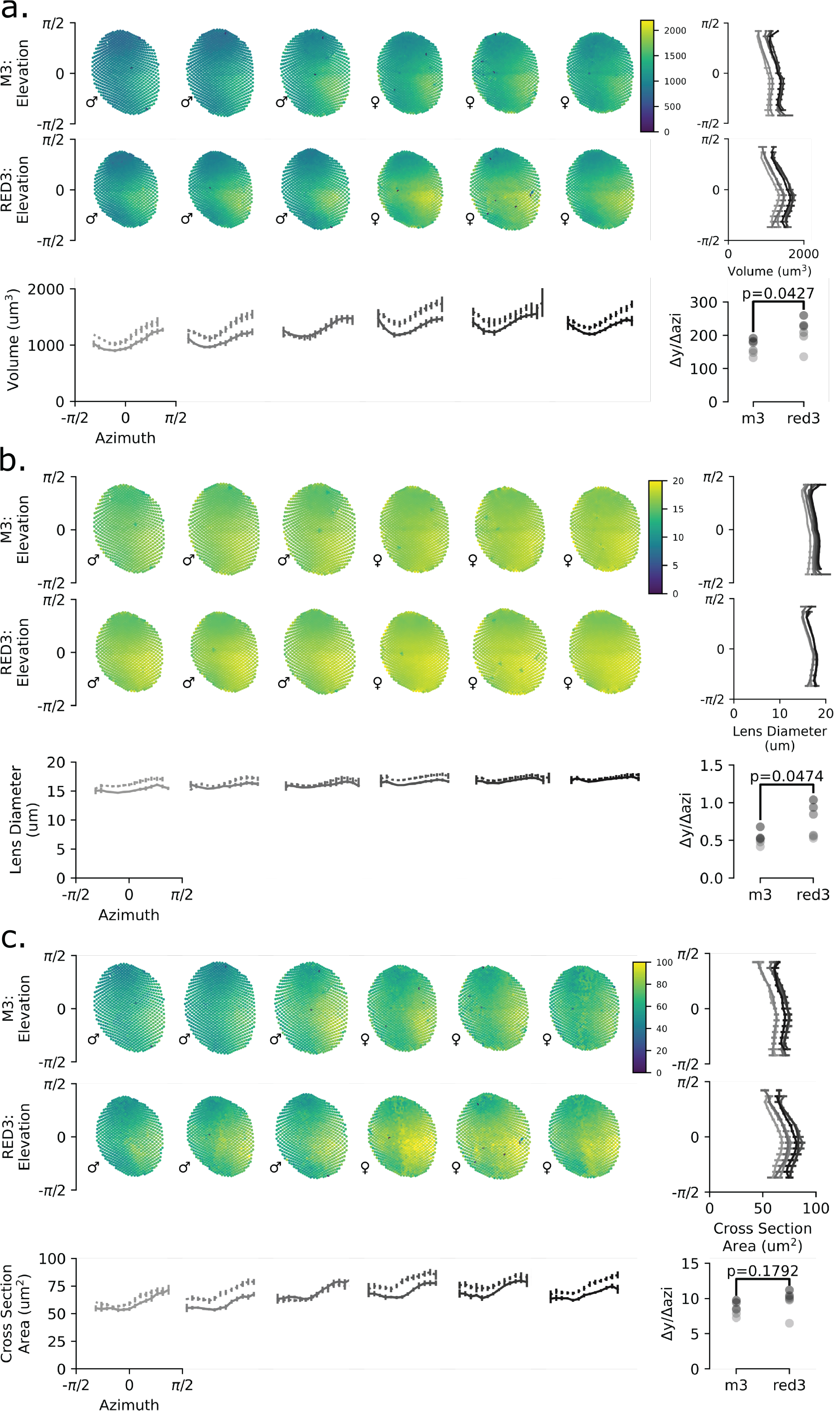

Supplement: Supplementary file 6 — Additional file 6: Fig. S5. Eye maps of ommatidia measurements across eyes of D. simulans and D. mauritiana. Eye maps of ommatidial lens volumes (a.), lens diameters (b.), and cross-sectional areas (c.), with their elevation (right column of each panel) and azimuthal (bottom row of each) profiles, and their azimuthal slope (bottom right inset of each) from 6 flies from each of the two species, D. mauritiana (RED 3) and D. simulans (M3), 3 males and females for each. The eyes are sorted in order from smallest to largest eye surface area, which also resulted in ordering by sex because males are generally smaller. Each dot of the scatter plot represents the location of an individual ommatidium in polar coordinates coloured by its 3D volume according to the colour bars. Line colours in the azimuthal and elevation profiles and dot colours in the azimuthal slope plots indicate the fly’s rank in order of eye size per species, such that the darkest one is the largest eye of that species. Each outcome is divided into 20 evenly spaced bins of elevation (line plots to the right) and azimuth (line plots below) with error bars indicating 3 times the standard error of the mean of each bin. Ordinary least squares was used to regress each outcome on azimuthal position to estimate and compare the azimuthal slope between the two species. Scatterplots in the bottom right show the resulting slope coefficients from those models. Note that azimuth here is in radians but was converted to degrees for the plots Fig. 3 and the calculation of the slope. a. Note that this presents the full dataset used in the elevation profiles of Fig. 3b and the azimuthal slopes in Fig. 3d. Data provided in Fig. 3_share.zip on figshare [29]. [file 12915_2024_1864_MOESM6_ESM.png]

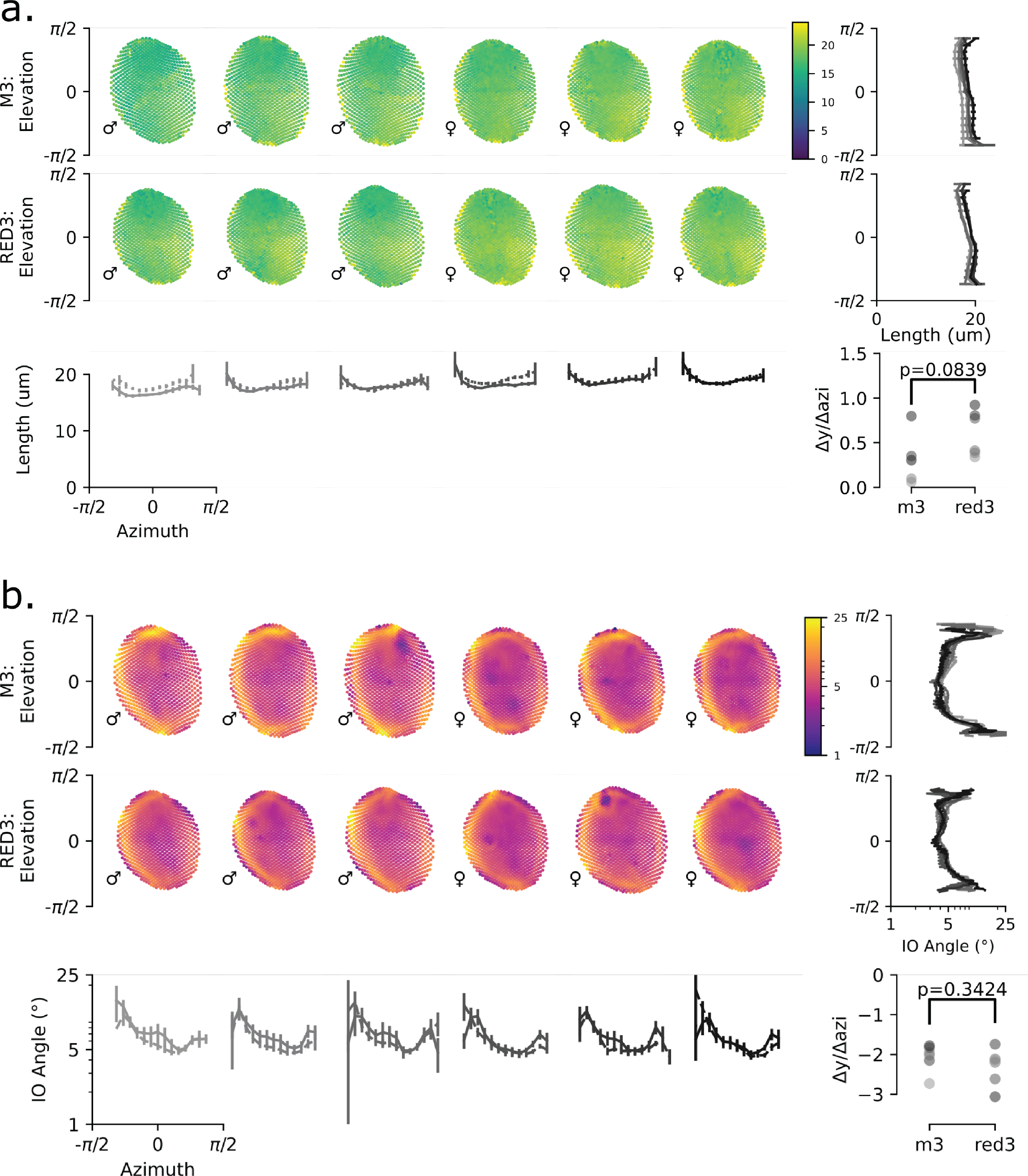

Supplement: Supplementary file 7 — Additional file 7: Fig. S6. Eye maps of ommatidia lens length and IO across eyes of D. simulans and D. mauritiana. a–b. Eye maps of ommatidial lens length (a.) and IO angle (b.) as in Suppl. Figure 5, with their elevation (right column of each panel) and azimuthal (bottom row of each) profiles, and their azimuthal slope (bottom right inset of each) from 6 flies from each of the two species, D. mauritiana (RED 3) and D. simulans (M3), 3 males and females for each. The eyes are sorted in order from smallest to largest eye surface area, which also resulted in ordering by sex because males are generally smaller. Each dot of the scatter plot represents the location of an individual ommatidium in polar coordinates coloured by its 3D volume according to the colour bars. Line colours in the azimuthal and elevation profiles and dot colours in the azimuthal slope plots indicate the fly’s rank in order of eye size per species, such that the darkest one is the largest eye of that species. Each outcome is divided into 20 evenly spaced bins of elevation (line plots to the right) and azimuth (line plots below) with error bars indicating 3 times the standard error of the mean of each bin. Ordinary least squares was used to regress each outcome on azimuthal position to estimate and compare the azimuthal slope between the two species. Scatterplots in the bottom right show the resulting slope coefficients from those models. Note that azimuth here is in radians but was converted to degrees for the plots Fig. 3 and the calculation of the slope. b. Note that, as in Fig. 3, the elevation profile for IO angle was plotted differently because plotting the binned averages obfuscates the horizontal band of high acuity along the equator, likely due to the large range of IO angles along azimuth. This also presents the full dataset used in Fig. 3f and the azimuthal slopes in Fig. 3h. Data provided in Fig. 3_share.zip on figshare [29]. [file 12915_2024_1864_MOESM7_ESM.png]

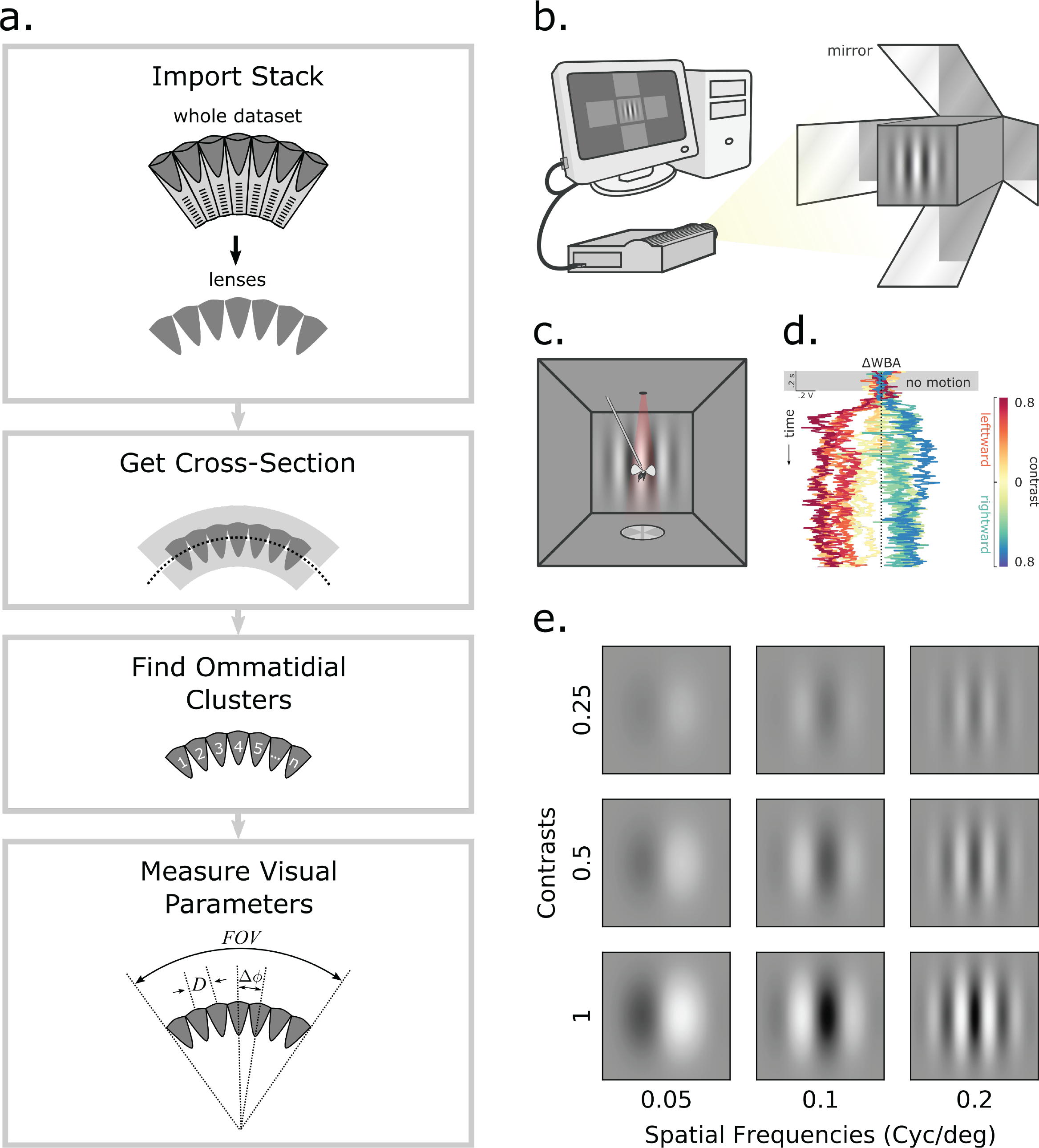

Supplement: Supplementary file 9 — Additional file 9: Fig. S7. Workflow for modelling and testing vision in Drosophila. a. Optical performance was evaluated across the visual field of each eye by applying the ODA-3D, which requires first prefiltering the stack to just its corneal lenses to fit a cross-sectional surface, apply a clustering algorithm, and finally take optically relevant measurements for each lens. b. Optomotor performance was evaluated by a virtual reality flight simulator using an open-source computer graphics library, high-speed projector, and precisely positioned first-surface mirrors to project high resolution and contrast stimuli surrounding 5/6 of the flies’ FOV. c. Flies were glued to a thin tungsten rod and centred within an acrylic cube lined with rear-projection material immersing them in the projection as in b. An IR light casted the shadow of each wing onto photodiodes below the fly designed to output the amplitude of each wingbeat shadow as a 1000 Hz voltage signal. The difference between the left and right wingbeat amplitudes (ΔWBA) is proportional to yaw torque and indicates the fly’s steering effort. d. We plotted the ΔWBA time series for an exemplary fly in response to 9 gratings of different contrast (corresponding to the line’s saturation) moving to the left or right (warm vs. cool hue), drawn from(Currea et al., 2022). Notice that the strength of the response is partially dependent on contrast while the direction corresponds generally to the direction of motion. Leftward motion ΔWBA responses were averaged with the inverse of rightward motion ΔWBA responses to account for directional biases in our measurement. These averages were then normalised to the maximum mean response per fly and averaged across each group to make the colormaps in Fig. 4. For each fly, an average of these normalised responses was taken from 0.5–1.25 s and used for plotting and comparing means in the bottom subplots (Fig. 4 c, f, and i). e. Sinusoidal moving gratings were used because they are [file 12915_2024_1864_MOESM9_ESM.png]
