# Supplementary material for: Evolution of compound eye morphology underlies differences in vision between closely related Drosophila species
Source: BMC Biol. 2024 Mar 19;22:67. doi: 10.1186/s12915-024-01864-7 (PMC10953123; doi:10.1186/s12915-024-01864-7)
Supplement: Supplementary file 8 — Additional file 8: Table S2. Parameters of the linear regression models of the allometries of optical parameters with respect to eye surface area. Each outcome was modelled as a linear combination of eye area and species (dummy-coded) with a constant intercept using ordinary least squares regression. The coefficient of determination (R2) and F-statistic are provided as measurements of goodness-of-fit with asterisks indicating the significance according to the key at the bottom of the table. The intercept and slope are the resulting coefficients of the regression model. D. mauritiana RED3 – D. simulans M3 is the pairwise difference of means after accounting for differences in eye size, such that values < 0 imply that D. simulans M3 values were greater than D. mauritiana RED3 relative to eye size. The significance of these statistics (F, intercept, slope, and D. mauritiana RED3 – D. simulans M3) is signified by the number of asterisks next to these values according to the key at the bottom of the table. [file 12915_2024_1864_MOESM8_ESM.pdf]

Table S2: Parameters of the linear regression models of the allometries of optical parameters with respect to eye surface area. Each outcome was modelled as a linear combination of eye area and species (dummy-coded) with a constant intercept using ordinary least squares regression. The coefficient of determination (R^2) and F-statistic are provided as measurements of goodness-of-fit with asterisks indicating the significance according to the key at the bottom of the table. The intercept and slope are the resulting coefficients of the regression model. *D. mauritiana* RED3 – *D. simulans* M3 is the pairwise difference of means after accounting for differences in eye size, such that values < 0 imply that *D. simulans* M3 values were greater than *D. mauritiana* RED3 relative to eye size. The significance of these statistics (F, intercept, slope, and *D. mauritiana* RED3 – *D. simulans* M3) is signified by the number of asterisks next to these values according to the key at the bottom of the table.

**outcome**	**R^2**	**F**	**intercept**	**slope**	**RED3 – M3**
lens count	0.96	110***	976***	2.4×10^{-3}***	-58***
lens diameters (μm)	0.97	159***	16***	2.1×10^{-5}***	0.54***
lens diameter IQR (μm)	0.73	12**	1.1***	-3.4×10^{-6}*	0.33**
median IO angle (°)	0.78	16**	4.9***	-7.1×10^{-6}***	-0.009
median equatorial IO angle (°)	0.92	53***	4.4***	-7.3×10^{-6}***	0.22***
IO angle IQR (°)	0.65	8**	3.1***	-2.2×10^{-6}	-0.51**
**<u>key:</u>**					
*: p ≤ .05, **: p ≤ .01, ***: p ≤ .001					
